# The role of ethical values and leadership commitment in fostering healthy work environments: implications for employee well-being and organizational sustainability

**DOI:** 10.3389/fpsyg.2026.1836495

**Published:** 2026-05-22

**Authors:** Lurdes Neves, Barbara Sousa, Tania Gaspar

**Affiliations:** 1 Instituto Superior de Gestão, CIGEST, Lisbon, Portugal; 2 CEFAGE – Universidade de Évora, Evora, Portugal; 3 Digital Human-Environment Interaction Labs (HEI-LAB), Lusófona University, Lisbon, Portugal; 4 Institute of Environmental Health (ISAMB), Lisbon, Portugal; 5 Aventura Social – Associação, Lisbon, Portugal

**Keywords:** burnout, leadership, mental health, organizational culture, psychosocial risks, values, workplace

## Abstract

In a rapidly changing labour market shaped by technological, social, and economic transformations, organizations must adapt their leadership practices to promote healthy and sustainable work environments. This study examines the relationship between organizational ethics and values, leadership engagement, and the psychosocial work environment. The sample included 4,551 workers from multiple sectors, of whom 63.1% were female, aged between 18 and 73 years (M = 44.8; SD = 10.80). Data were collected using a short version of the Healthy Workplace Ecosystems Instrument (EATS), based on the Healthy Workplaces model proposed by the World Health Organization. Group comparisons identified higher-risk profiles, including employees experiencing burnout symptoms, chronic illness, unstable employment conditions, or dissatisfaction with remuneration. Mediation analysis revealed that the relationship between leadership engagement and the psychosocial work environment is mediated by organizational ethics and values, highlighting the importance of ethical leadership and organizational culture in promoting employee well-being and reducing psychosocial risks.

## Introduction

The contemporary workplace is undergoing profound transformations driven by technological advancements, evolving workforce expectations, and increasing recognition of employee well-being as a strategic organizational priority. In this context, fostering healthy work environments has become essential for both organizational sustainability and employee mental health (Backhaus et al., 2024; Pavlista et al., 2024). Recent research highlights the growing relevance of psychosocial risks in modern organizations, particularly in contexts marked by organizational change, digitalization, and increased work demands.

The World Health Organization’s Healthy Workplaces framework (World Health Organization, 2010) provides a comprehensive model for understanding how organizational conditions influence employee well-being, emphasizing the importance of psychosocial risk prevention. Within this framework, ethical values, organizational culture, and leadership practices emerge as critical determinants of healthy work environments (Alali et al., 2025).

Ethical leadership has been widely conceptualized as the demonstration of normatively appropriate conduct through personal actions and interpersonal relationships, promoting such conduct among followers through communication, reinforcement, and decision-making (Brown and Treviño, 2006). More recent evidence reinforces that ethical leadership is positively associated with employee well-being, reduced emotional exhaustion, and enhanced organizational functioning, particularly through mechanisms such as psychological safety and affective commitment (Santiago-Torner et al., 2024; Amory et al., 2024). Furthermore, ethical leadership has been shown to influence employee attitudes and behaviors through psychological capital and moral identity processes, highlighting its multidimensional and dynamic nature (Ahmed and Khan, 2024).

Simultaneously, organizational values serve as guiding principles that shape decision-making processes, behavioral norms, and institutional practices. When aligned with leadership behaviors, these values foster a coherent organizational culture that supports fairness, transparency, and employee well-being. Evidence suggests that such value-based cultures can mitigate the negative effects of psychosocial risks, particularly in demanding and uncertain work environments (Backhaus et al., 2024).

Despite these advances, existing research has largely examined leadership, ethics, and psychosocial work environments in isolation or through direct-effect models. Less attention has been given to the underlying mechanisms through which leadership influences psychosocial outcomes. In particular, the mediating role of organizational ethics and values remains underexplored, especially in non-Anglo-Saxon contexts, where organizational dynamics and cultural influences may differ significantly.

Furthermore, the post-pandemic shift toward hybrid and remote work arrangements has introduced new challenges and opportunities, reinforcing the need to understand how ethical leadership and organizational values operate in increasingly flexible, digital, and decentralized work environments (Santiago-Torner et al., 2024).

Addressing these gaps, the present study aims to examine the relationships between leadership engagement, organizational ethics and values, and psychosocial work environment. Specifically, it proposes a mediation model in which ethics and values function as a key explanatory mechanism linking leadership to psychosocial outcomes.

By integrating ethical leadership theory with psychosocial risk frameworks, this study contributes to the literature by offering a process-based understanding of how leadership translates into employee well-being. Additionally, by focusing on a large multi-sector sample within the Portuguese context, this research provides empirical evidence from an underrepresented setting in organizational psychology research.

*H1*: Ethics and values are positively associated with psychosocial work environment.

*H2*: Leadership engagement is positively associated with psychosocial work environment.

*H3*: Leadership engagement is positively associated with ethics and values.

*H4*: Ethics and values mediate the relationship between leadership engagement and psychosocial work environment.

## Materials and methods

### Design and participants

This study adopted a cross-sectional design, using a non-probabilistic convenience sample, which allows for the examination of associations between variables at a single point in time. While appropriate for identifying relationships between constructs, this design does not permit causal inferences.

A total of 4,551 participants from multiple organizational sectors in Portugal were included in the study. Of these, 63.1% were female (*n* = 2,795), with ages ranging from 18 to 73 years (M = 44.8, SD = 10.80).

Participants were recruited through organizations affiliated with the Portuguese Laboratory for Healthy Workplaces (LABPATS). A total of 50 organizations were invited to participate, of which 40 completed data collection within the study timeframe. These organizations represented diverse sectors, including public administration, healthcare, education, social services, retail, and banking.

While this strategy enabled access to a large and diverse sample, it may also introduce selection bias, as participating organizations may have a greater interest in workplace well-being, psychosocial risk management, and ethical practices (Backhaus et al., 2024). Therefore, caution is warranted when generalizing the findings.

### Procedure

Data were collected through an online survey distributed internally by participating organizations. Prior to participation, individuals were informed about the study’s objectives, confidentiality, anonymity, and voluntary nature. Informed consent was obtained electronically before access to the questionnaire.

The study was approved by the Ethics Committee of the Hospital of Espírito Santo of Évora, ensuring compliance with ethical standards for research involving human participants.

### Measures

The study used selected dimensions of the Healthy Work Environments Ecosystems Instrument (EATS), a validated tool based on the WHO Healthy Workplaces model (World Health Organization, 2010).

Three dimensions were included, based on theoretical alignment with the study objectives:

- Ethics and Values (3 items; *α* = 0.91)
- Leadership Engagement (3 items; α = 0.95)
- Psychosocial Work Environment (6 items; α = 0.91)

Additionally, burnout symptoms were assessed using a 3-item scale (α = 0.86).

All items were measured on a 5-point Likert scale. Internal consistency values indicate good reliability across all constructs.

The selection of these dimensions was theoretically driven; however, excluding the remaining EATS dimensions may limit construct comprehensiveness. This limitation is acknowledged in the Discussion section.

### Data analysis

Descriptive and correlational analyses were conducted to examine relationships among variables. Group comparisons were performed using t-tests and ANOVA.

Mediation analysis was conducted using PROCESS macro (Model 4; Hayes, 2018), with 5,000 bootstrap samples. Indirect effects and corresponding 95% confidence intervals were computed to assess the mediating role of Ethics and Values.

To assess common method variance, Harman’s single-factor test was performed. Results indicated that a single factor did not account for the majority of variance, suggesting that common method bias is unlikely to substantially affect the findings.

Given the cross-sectional design, all findings are interpreted as associative rather than causal.

## Results

The sample included 4,551 participants, the majority (63.1%) of whom were female (*n* = 2,795), aged between 18 and 73 (M = 44.79, SD = 10.80). Most of the participants (76.2%) revealed having burnout symptoms (*n* = 3,468). Table 1 shows the Sociodemographic characteristics. The correlations were all significant. A positive, strong, and significant association was observed between Ethics & Values (EV) and Engagement to Leadership (EL) (r = 0.880, *p* = 0.000). There were two positive, moderate, and significant associations between EL and Psychosocial Work Environment (PWECL) (r = 0.638, *p* = 0.000) and between EV and PWECL (r = 0.623, *p* = 0.000) (Table 2).

**Table 1:** Sociodemographic characteristics.

Variables		*%* or *M ± SD*
Fri (*n* = 4551)	Female	63.1 (2795)
	Male	36.9 (1636)
Age (*n* = 4546)		Min = 18; Max = 73 44.79 ± 10.80
Burnout symptoms (4551)	In	23.8 (1081)
	Yes	76.2 (3468)

**Table 2:** Correlations.

	EV	EL	PWECL
Ethics & values (EV)		0.880***	0.623***
Engagement to leadership (EL)			0.638***
Psychosocial work environment (PWECL)			

***indicates statistical significance at *p* < 0.001.

Table 3 shows the comparison of groups related to burnout symptoms, work contract, remuneration satisfaction, and chronic illness in each of the variables. Regarding burnout symptoms, statistically significant differences were found. Participants who did not show burnout symptoms had higher scores on the EV, EL, and PWECL.

**Table 3:** Comparison of groups.

	Descriptive statistics				Significance tests and effect size
	$\underline{x}$	**SD**	$\underline{x}$	**SD**	
	Without burnout symptoms		With burnout symptoms		
Ethics & values (EV)	**3.53**	0.90	3.20	0.91	**t (1809.875) = 10.538, *p* < 0.001, d = 0.91**
Engagement to leadership (EL)	**3.73**	0.94	3.29	0.95	**t (1812.889) = 13.308, *p* < 0.001, d = 0.95**
Psychosocial work environment (PWECL)	**3.55**	1.10	3.18	0.93	**t (1590,650) = 9,884, *p*<0.001, d = 0.98**
	Fixed-term work contract		Stable work contract		
Ethics & values (EV)	3.23	0.92	**3.34**	0.91	**t (4542) = −3.891, *p* < 0.001, d = 0.92**
Engagement to leadership (EL)	3.37	0.96	**3.43**	0.98	**t (4544) = −2.210, *p* = 0.027, d = 0.97**
Psychosocial work environment (PWECL)	3.25	0.98	3.30	1.00	t (4544) = −1.525, *p* = 0.127, d = 0.98
	Not satisfied remuneration		Satisfied remuneration		
Ethics & values (EV)	3.10	0.91	**3.63**	0.83	**t (3252.763) = −19.523, *p* < 0.001, d = 0.88**
Engagement to leadership (EL)	3.20	0.96	**3.78**	0.86	**t (3313.018) = −20.672, *p* < 0.001, d = 0.93**
Psychosocial work environment (PWECL)	3.08	0.94	**3.66**	0.96	**t (4547) = −19.420, *p* < 0.001, d = 0.95**
	Without CI (CI = Chronic Illness)		With CI		
Ethics & values (EV)	**3.33**	0.91	3.15	0.93	**t (2481.561) = 6.010, *p* < 0.001, d = 0.92**
Engagement to leadership (EL)	**3.46**	0.95	3.23	1.00	**t (2422.562) = 7.131, *p* < 0.001, d = 0.96**
Psychosocial work environment (PWECL)	**3.33**	0.98	3.12	0.99	**t (4546) = 6.714, *p* < 0.001, d = 0.98**

In terms of the work contract, statistically significant differences were found in EV and El, in the sense that those who have a stable work contract have higher values in the variables mentioned. No statistically significant differences were found for PWECL. As for satisfaction with pay, statistically significant differences were found in all the variables. In the sense that those who reported being satisfied with their remuneration had higher values for EV, EL, and PWECL.

As for chronic illness, statistically significant differences were found for all the variables, in the sense that participants who said they have no chronic illness had higher values for EV, EL, and PWECL.

Table 4 shows the group comparisons, for generations, teleworking, and educational level, by ANOVA.

**Table 4:** Comparison of groups.

	$\underline{x}$ (SD)	$\underline{x}$ (SD)	$\underline{x}$ (SD)	$\underline{x}$ (SD)	Significance test and effect size
	Generation Z	Generation Y	Generation X	Baby Boomers	
Ethics & values (EV)	**3.51 (0.89)**	3.25 (0.94)	3.26 (0.91)	3.27 (0.88)	**F (3, 4,542) = 6.081, *p* < 0.001**
Engagement to leadership (EL)	**3.65 (0.90)**	3.40 (0.98)	3.37 (0.96)	3.36 (0.97)	**F (3, 4,544) = 6.794, *p* < 0.001**
Psychosocial work environment (PWECL)	**3.55 (0.97)**	3.26 (0.99)	3.26 (0.99)	3.20 (0.99)	**F (3, 4,544) = 7.685, *p* < 0.001**
	No remote working	Remote working			
Ethics & values (EV)	3.21 (0.94)	**3.52 (0.77)**			**F (2, 4,546) = 34.339, *p* < 0.001, η = 0.015**
Engagement to leadership (EL)	3.34 (0.99)	**3.62 (0.83)**			**F (2, 4,548) = 24.792, *p* < 0.001, η = 0.011**
Psychosocial work environment (PWECL)	3.17 (1.00)	**3.58 (0.84)**			**F (2, 4,548) = 73.881, *p* < 0.001, η = 0.031**
	Compulsory schooling	Bachelor’s degree			
Ethics & values (EV)	**3.37 (0.91)**	3.24 (0.92)			**F (2, 4,544) = 12.805, *p* < −001**
Engagement to leadership (EL)	**3.48 (0.94)**	3.35 (0.99)			**F (2, 4,546) = 9.498, *p* < 0.001**
Psychosocial work environment (PWECL)	3.31 (0.97)	3.20 (1.01)			**F (2, 4,546) = 8.654, *p* < 0.001**

Bold values indicate the highest mean scores across groups.

As for the generations, statistically significant differences were found for all the variables. Generation Z revealed higher values for EV, EL, and PWECL.

In terms of teleworking, statistically significant differences were found for all the variables. Participants who work remotely show higher values for EV, EL, and PWECL. Statistically significant differences were found for all the variables in terms of schooling. Participants with only compulsory education showed higher levels of EV and El, while participants with postgraduate training showed higher values in PWECL.

To find out whether Ethics mediates the relationship between Leadership (independent variable) and Psychosocial Work Environment (dependent variable), a mediation analysis was carried out using PROCESS, with a sample of 4,546 participants.

The first model of the analysis tested the effect of Leadership on Ethics (mediator). The results indicated that Leadership significantly predicts Ethics (b = 0.8798, *p* < 0.001). The coefficient of determination (R^2^ = 0.7741) indicates that 77.41% of the variance in Ethics can be explained by Leadership.

**Figure 1:**
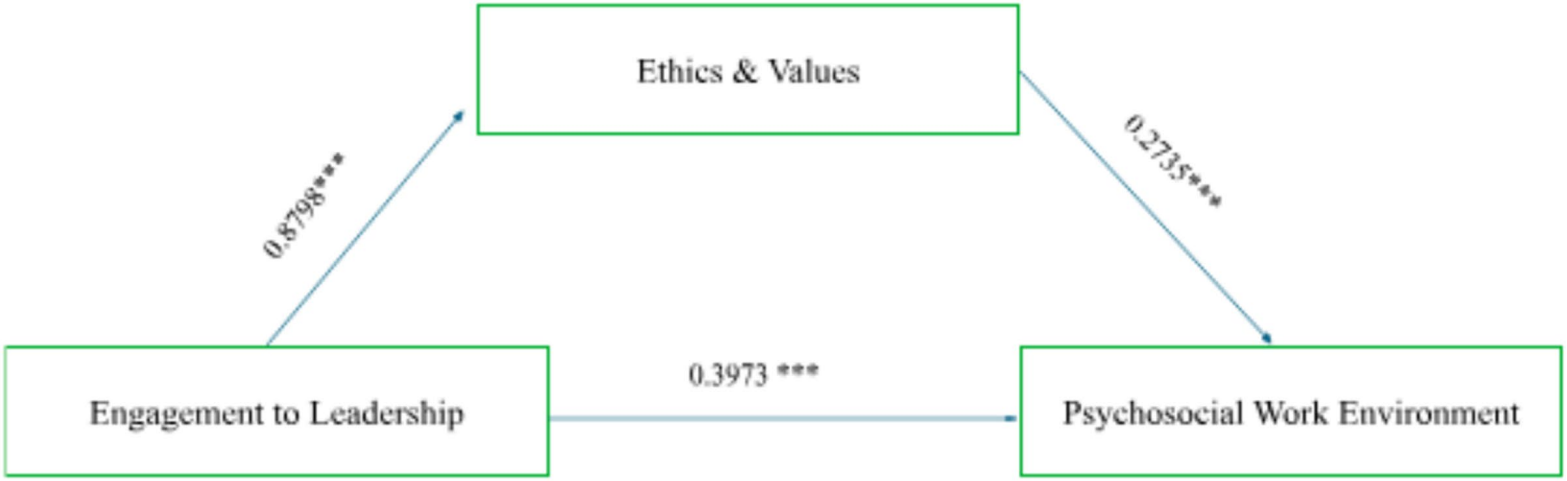
Mediation model.

The second model analyzed the simultaneous effects of Leadership and Ethics on Psychosocial Work Environment. The results indicated that both Leadership (b = 0.3973 *p* < 0.001) and Ethics (b = 0.2735, *p* < 0.001) significantly predict Psychosocial Work Environment. The coefficient of determination (R^2^ = 0.4238) indicates that 42.38% of the variance in Psychosocial Work Environment can be explained by Leadership and Ethics. Figure 1 shows the mediation model.

In conclusion, significant positive correlations were found between Ethics & Values (EV), Leadership Engagement (LE), and Psychosocial Work Environment (PWECL): EV and LE (r = 0.880, *p* < 0.001), EV and PWECL (r = 0.623, *p* < 0.001), and LE and PWECL (r = 0.638, *p* < 0.001).

Group comparisons revealed that workers with burnout symptoms, unstable contracts, lower satisfaction with remuneration, or chronic illnesses scored significantly lower in all three dimensions (*p* < 0.001). Generation Z and teleworkers scored higher on ethical values, leadership engagement, and psychosocial safety.

Mediation analysis confirmed that Ethics & Values significantly mediate the relationship between Leadership and Psychosocial Work Environment. Leadership predicted Ethics (b = 0.8798, *p* < 0.001), and both predicted PWECL (LE: b = 0.3973; EV: b = 0.2735; *p* < 0.001).

## Discussion

The present study aimed to examine the relationships between leadership engagement, organizational ethics and values, and psychosocial work environment, as well as to identify groups at higher risk of adverse psychosocial outcomes.

The findings indicate that leadership engagement, ethics and values, and psychosocial work environment are positively associated. Notably, ethics and values emerged as a significant mediating mechanism, suggesting that leadership influences psychosocial conditions through the promotion of ethical organizational cultures. This finding aligns with recent research emphasizing the role of ethical leadership in shaping employee well-being through relational and cognitive mechanisms (Santiago-Torner et al., 2024; Ahmed and Khan, 2024).

These results contribute to the literature by advancing a process-based understanding of leadership effects, highlighting that leadership alone may not directly shape employee well-being, but rather operates through the institutionalization of ethical values and practices. This perspective is consistent with emerging evidence suggesting that leadership effectiveness is contingent upon organizational context and value alignment (Hassan et al., 2023).

The identification of higher-risk groups—such as employees experiencing burnout symptoms, chronic illness, lower remuneration satisfaction, or precarious employment—underscores the importance of targeted organizational interventions, particularly in contexts characterized by increasing psychosocial demands and organizational change (Backhaus et al., 2024).

The high prevalence of burnout symptoms observed in this study should be interpreted cautiously. As the measure reflects self-reported symptoms rather than clinical diagnosis, it may overestimate actual prevalence. Nonetheless, it signals a concerning trend that warrants organizational attention and intervention (Alali et al., 2025).

The strong association between ethics and leadership also raises important conceptual considerations. While consistent with theoretical expectations, it suggests the need for further refinement of measurement models to ensure discriminant validity between constructs and to better capture their distinct contributions to organizational outcomes.

## Limitations

This study presents several limitations. First, the use of a cross-sectional design precludes causal inferences. Second, the reliance on self-reported data collected through a single instrument raises the possibility of common method bias, although statistical tests suggest this is not a major concern.

Third, the use of a convenience sample based on organizational affiliation with LABPATS may limit generalizability. Participating organizations may already demonstrate greater awareness of workplace well-being and ethical practices.

Finally, the use of only selected dimensions of the EATS instrument may limit construct comprehensiveness.

## Conclusion

This study highlights the central role of ethical values in translating leadership engagement into healthier psychosocial work environments. By demonstrating the mediating function of organizational ethics, the findings provide a more nuanced understanding of how leadership contributes to employee well-being.

Promoting ethical organizational cultures, aligned with leadership practices, emerges as a key strategy for reducing psychosocial risks and fostering sustainable and healthy workplaces.

Future research should adopt longitudinal designs, more comprehensive measurement models, and cross-cultural approaches to further advance this field, particularly in the context of evolving work arrangements and increasing organizational complexity.

### Future research directions

Future studies should explore the longitudinal effects of ethical leadership and organizational values on psychosocial outcomes across diverse cultural and economic contexts. Investigating how digital transformation and hybrid work environments reshape leadership dynamics and ethical culture will be crucial. Additionally, qualitative research approaches may deepen understanding of employee perceptions of ethical climates and their lived experiences of psychological safety.

Comparative cross-national studies could further illuminate how cultural dimensions mediate the relationship between leadership ethics and well-being. Finally, future research should address interventions that build ethical competencies in leaders and assess their impact on organizational resilience and sustainability.

The study illustrates the fundamental importance of organizational culture and leadership in promoting the well-being of workers and strategies to reduce psychosocial risks at work, we suggest the following measures:

Promote an organizational culture with fair and transparent ethical values, centering all policies on individual and organizational well-being.

Promote healthy leadership, with empathetic leadership skills and promoters of psychological safety.

Involve leaders and professionals in the design of priorities and measures for the promotion of well-being and organizational performance.

Provide the available means to promote the individual health of workers, namely, well-being initiatives, physical activity programs and psychological support, leadership coaching, highlighting the importance of a holistic approach to health in the workplace (Chapman, 2003; Gaspar et al., 2023).

## Data Availability

The original contributions presented in the study are included in the article/supplementary material, further inquiries can be directed to the corresponding author.
